# The complete mitochondrial genome of the White-Nose Syndrome pathogen, *Pseudogymnoascus destructans*

**DOI:** 10.1080/23802359.2017.1280706

**Published:** 2017-02-02

**Authors:** Adrian Forsythe, Jianping Xu

**Affiliations:** Department of Biology, McMaster University, Hamilton, Canada

**Keywords:** White-Nose Syndrome, mitochondrial genome, *Pseudogymnoascus destructans*

## Abstract

In this study, the complete mitochondrial genome of the White-Nose Syndrome pathogen, *Pseudogymnoascus destructans* (=*Geomyces destructans*), is sequenced. The circular mitochondrial genome is 32,181 bp long and encodes 13 standard proteins, 2 ribosomal RNA subunits, and 27 tRNAs. The genome contains two introns located within the cytochrome c oxidase subunit 1 gene (*cox1*) and the large ribosomal RNA subunit (*rrnl*), with each intron encoding one gene, *orf110* and *rps3*, respectively. Phylogenetic analysis of the concatenated mitochondrial protein-coding genes of *P. destructans* and close representatives in Leotiomycetes showed that *P. destructans* was closely related to *Pseudogymnoascus pannorum*, consistent with nuclear genes.

The White-Nose Syndrome (WNS) is a devastating infection of North American bats, caused by the fungal pathogen, *Pseudogymnoascus destructans* (Minnis & Lindner 2013). The infection is characterized by a mycosal infection of epithelial tissues (Meteyer et al. 2009), which eventually leads to electrolyte imbalance, evaporative water loss, frequent arousal from hibernation (Willis et al. 2011), and starvation (Cryan et al. 2010). Since 2006, the WNS epidemic has spread to 29 US States and 5 Canadian Provinces, with over 6 million cases of bat mortality attributed to *P. destructans* (US Fish and Wildlife Service 2016).

The data published on *P. destructans* genomes by Cuomo et al. (2010) and Drees et al. (2016) contained incompletely assembled and un-annotated mitochondrial DNA sequences. Here, we present the complete mitochondrial genome sequence of *P. destructans* strain 20631-21, the initial case of WNS was from William’s Hotel Mine in New York, USA (Blehert et al. 2009). This sample was collected bat by the US Forestry Service from a deceased little brown in 2008 (ATTC: MYA-4855). We used an Illumina MiSeq platform to sequence the full genome of *P. destructans* and mitochondrial sequences were extracted from our raw reads through *in silico* baiting, trimmed for quality using MIRA (V 4.0.2) (Hahn et al. 2013), and merged overlapping paired end reads using FLASh (V 1.2.11) (Magoč & Salzberg 2011). The complete mitochondrial sequence was assembled using the MITObim pipeline (V 1.8) (Hahn et al. 2013), yielding a circular molecule of 32,182 bp, with a GC content of 28.5%. The genome was built based on 46,037 paired-end reads with an average quality of 84 and an average coverage 510×.

The mitochondrial genome was annotated through the MITOS web server, with 16 predicted open reading frames (ORFs) which overlapped with functional proteins (Wheeler et al. 2003; Camacho et al. 2008; Bernt et al. 2013) (Figure S1). The full mitochondrial genome encodes 13 genes of the oxidative phosphorylation system, the small and large ribosomal RNA subunits (*rns* and *rnl* respectively), and 27 tRNA genes (GenBank Accession: KY318514.1). A 112 amino acid fragment of the 5′ end of the *cox1* gene (labeled as *cox1-1*) is separated from the rest of cox1 (labeled as *cox1-2*) by a 1330 bp intron, itself containing the intronic gene *orf110*, encoding the protein domain for catalytic GIY-YIG and putative intron-encoded endonuclease bI1. An additional intron was found within the large ribosomal RNA subunit, which codes for ribosomal protein S3 (*rps3*). A 122bp fragment of the *atp9* gene was found within *P. destructans* mitochondria, although this sequence was not found within the *P. pannorum* mitochondrial genome (Zhang et al. 2016).

We also present the phylogenetic relationships among 12 representative sequenced filamentous ascomyceteous species (Figure 1), based on concatenated nucleotide sequences of 13 mitochondrial protein-coding genes aligned using MAFFT (V 7.205) (Katoh & Standley 2013). The phylogenetic analysis was completed using a Maximum Likelihood approach with a GTRGAMMA model of nucleotide substitution and rate heterogeneity with RAxML (V 8.0.25) (Stamatakis 2014) and visualized using the R package ggtree (Yu et al. 2016).

**Figure 1.**
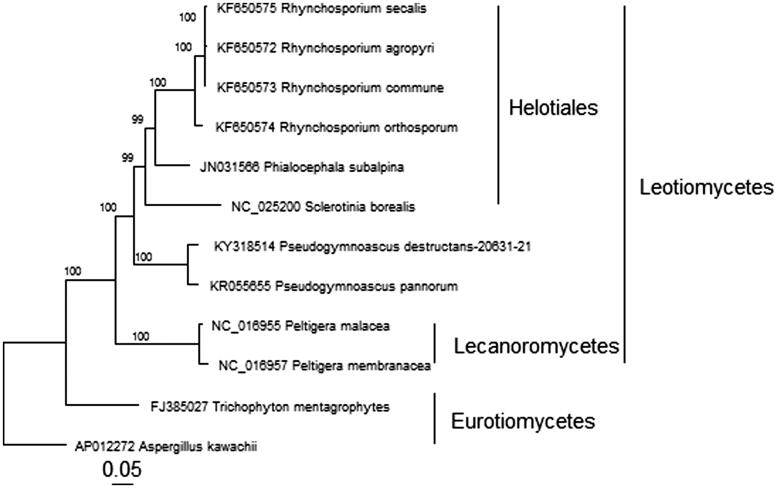
Phylogenetic relationship between *P. destructans* and representatives of related ascomycete species based on concatenated nucleotide sequences of 13 protein-coding genes: *atp6*, *atp8*, *cob*, *cox1*, *cox2*, *cox3*, *nad1*, *nad2*, *nad3*, *nad4*, *nad4L*, *nad5*, and *nad6* for a total of 10,372 characters. All non-*P. destructans* sequences were obtained from GenBank with accession numbers shown before the species names.
